# Severity of Child Autistic Symptoms and Parenting Stress in Mothers of Children with Autism Spectrum Disorder in Japan and USA: Cross-Cultural Differences

**DOI:** 10.1155/2022/7089053

**Published:** 2022-07-12

**Authors:** Noriko Porter, Katherine A. Loveland, Sepideh Saroukhani, Yana Posey, Kana Morimoto, Mohammad H. Rahbar

**Affiliations:** ^1^Department of Human Development, Washington State University, Pullman 99164, USA; ^2^Louis A Faillace MD Department of Psychiatry and Behavioral Sciences, University of Texas Health Science Center at Houston/McGovern Medical School, Houston 77054, TX, USA; ^3^Division of Clinical and Translational Sciences, Department of Internal Medicine, McGovern Medical School, The University of Texas Health Science Center at Houston, Houston, TX 77030, USA; ^4^Biostatistics/Epidemiology/Research Design (BERD) Component, Center for Clinical and Translational Sciences (CCTS), The University of Texas Health Science Center at Houston, Houston, TX 77030, USA; ^5^Department of Psychiatry, Graduate School of Medicine, Kyoto University, Kyoto 606-8507, Japan; ^6^Department of Epidemiology, Human Genetics, and Environmental Sciences (EHGES), School of Public Health, The University of Texas Health Science Center at Houston, Houston, TX 77030, USA

## Abstract

The purpose of this study was to compare the relationship between parenting stress and autistic symptom severity in the U.S. and Japan. Fifty-two U.S. and 51 Japanese mothers of children aged 2–12 with autism completed measures of parenting stress and child characteristics, including the parenting stress index (PSI), the social communication questionnaire (SCQ), and social responsiveness scale-2 (SRS-2). There was a nonlinear relationship between the child's autistic symptom severity and parenting stress in both countries. We also found some cultural differences: in the parent domain, the relationships between children's SCQ scores and PSI scores differed significantly between the U.S. and Japan. Our findings suggest that autistic severity symptom scores may reflect cross-cultural differences in parenting beliefs, views toward autism, and response styles for evaluating children's behavior. The findings also suggest that parents need support regardless of the child's autism severity, including those with mild to moderate symptoms. Expanding on this line of research and understanding cultural influences on parenting stress may help service providers and agencies offer more culturally sensitive services, parent-education courses, and intervention programs.

## 1. Introduction

Autism (autism spectrum disorder or ASD) (DSM-5) is a neurodevelopmental condition that begins in early childhood and affects social communication and cognition. It is expressed along a spectrum of mild to severe symptoms. Autism is found worldwide, although the estimates of prevalence are only available in some countries. Internationally, the parents of children with autism have been found to experience a higher level of parenting stress than those who are raising typically developing children or children with other disabilities e.g., [1–8]. Numerous studies have examined contributors to parenting stress, including child and parent characteristics [1, 4, 9]. However, evidence supporting the relationship between child autistic symptom severity and parenting stress has been inconsistent [4, 10–12]. In addition, only a handful of studies have investigated the relationship between child autistic symptom severity and parenting stress in non-Western countries [8, 13, 14]. Although the symptoms of autism may be similar across different cultures, the meaning attributed to the symptoms may differ among parents from different cultural groups [15]. The present study aimed to compare the relationship between parenting stress in mothers of children with autism and child's autistic symptom severity in the U.S. and Japan.

In Japan, the prevalence of autism has been estimated to be 2.75% [16] according to the National Database [NDB] of 313,353 children, whereas in the U.S., it has been found to be one in 44 children according to Centers for Disease Control and Prevention (CDC) [17]. Although these countries share some common characteristics, such as a high rate of autism [16,17] and intensive research on autism [18], differences in cultural beliefs and social norms can result in distinctive differences in parenting [19]. Culturally, Japan is typically classified as collectivistic, and the U.S. is classified as individualistic [20]. Although Japan has been influenced by Western individualistic culture, an emphasis on collectivism remains. Japanese culture continues to strongly emphasize maintaining harmony and adjusting oneself to the expectations of the group or society [21,22].

Given the considerable differences in values and social conventions between the U.S. and Japan, it appears reasonable to expect that children's autistic behaviors that appear inconsistent with social norms may trigger more stress for Japanese parents than for U.S. parents. In cultures where a high value is placed on social harmony and not standing out from the crowd, such as Japan and China, there may be greater stigma and blame placed upon the child and parent for the child's inappropriate behavior, consequently leading to higher caregiver burdens [23–25]. For example, when Chinese parents of children with mental illnesses experience stigma in society, their face-saving concerns have been found to contribute to higher parenting stress levels [23].

Although researchers seem to agree on a positive association between parenting stress and children's problem behavior (child's internalizing and externalizing problem behaviors) [2, 4, 14, 26, 27], the findings related to the association between child autistic symptom severity and parenting stress have been inconsistent [4, 7, 9, 11, 12, 28, 29]. Some studies have found that parenting stress is influenced by the severity of autistic symptoms [28,30], while other studies found no association between these factors and parenting stress [4, 7, 12, 26, 27, 31]. These studies used various autism diagnostic tools to measure the severity of autistic symptoms, including scores derived from the social communication questionnaire (SCQ), the social responsiveness scale (SRS), and the autism behavior checklist (ABC).

Parenting stress studies conducted to examine the relationship between parenting stress and child autistic symptom severity in Japan have also been inconsistent. In Japanese families, a positive relationship between the child's core autism behaviors and parenting stress has been identified in some studies [32–34]. Asano et al. [35] found that autistic symptoms (social communication, social interaction, and restricted or repetitive patterns of behavior or interests) were positively related to the child domain of parenting stress (parenting stress originates from child temperament and characteristics) as measured by the PSI (parenting stress index) in mothers of preschool children with autism. However, mothers of children with “high functioning autism” showed higher parent domain parenting stress than mothers of children with “low functioning autism.” Asano hypothesized that this finding resulted from the invisible nature of autism for children who are intellectually able. Children who do not appear disabled but have unusual behaviors may be viewed by strangers as simply ill-behaved and their parents as unable to control them. If so, parents may feel stigmatized by others, and consequently, they may experience loneliness and anxiety. Similarly, Mori et al. [36] found higher parenting stress in parents of children with Asperger's syndrome than in parents of children with autistic disorder.

The present study aims to add to the international literature on parenting stress by comparing the differences in reported parenting stress and autistic symptom severity for mothers in the U.S. and Japan. Despite the vast amount of research on stress in parents of children with autism, to our knowledge, it is the first study that investigates cross-cultural differences in the relationship between parenting stress and autistic symptom severity. The study that has come the closest to approaching it was the one in the U.S. and Korea that compared the different ways parenting stress is related to child problem behaviors as measured by the child behavior checklist (CBCL) using a sample of clinically referred youth and their mothers [2]. In this study, Korean mothers reported significantly higher parenting stress measured by the Korean version of parenting stress index-short form but significantly lower childhood problem behaviors compared to U.S. mothers. In addition, a positive relationship was found between child problem behavior and parenting stress for the U.S. sample but not for the Korean sample. As seen in Chung's study, child behavior and symptoms may be interpreted by parents differently in different cultural groups, which subsequently influences parental mental health [15]. The purpose of this study is to compare the relationship between parenting stress and autistic symptom severity in the U.S. and Japan.

## 2. Materials and Methods

### 2.1. Participants

The participants included 52 U.S. mothers and 51 Japanese mothers who have a child with autism between ages 2 to 12 years. The inclusion criteria for parents included the following: (a) the child has received the formal diagnosis of autism by a qualified professional as reported by the mother, using various tests, either screening (e.g., parent-interview ASD rating scales-text revision (PARS-TR) [37], SRS-2 [38, 39], and SCQ [40, 41]) or diagnostic (autism diagnostic observation schedule™, second edition (ADOS-2) [42, 43] within a comprehensive evaluation, (b) the mother is of age 18 years and above, and (c) the mother and her parents were born in the U.S. or Japan. We included this last criterion to ensure cultural homogeneity. Information about the age and year of the child's formal diagnosis and the type of facility where the diagnosis was given were obtained from a background questionnaire completed by the parents. The participants were recruited from nine clinics (US = 6, Japan = 3), nine support organizations (US = 4, Japan = 5), nine parent support groups (US = 5, Japan = 4), five university social media sites (US = 3, Japan = 2), three schools for children with developmental disabilities (US = 2, Japan = 1), and three social media platforms (US = 1, Japan = 2). While these organizations provided some breadth in the sample that was recruited, it is possible that parents who are more educated about autism were over-represented, given that they were associated with these organizations.

### 2.2. Procedures and Measures

After signing the written consent form, all participants were asked to complete the following four questionnaires: a background questionnaire, the parenting stress index (PSI) [44], the social communication questionnaire (SCQ) [41], and the social responsiveness scale-2 (SRS-2) [38]. For the Japanese participants, we used a Japanese-translated consent form and background questionnaire. The Japanese version of the PSI, the SRS-2, and the SCQ were available through Japanese publishers.

This study was reviewed and approved by the Committee for the Protection of Human Subjects Internal Review Board of the University of Texas Health Science Center at Houston/McGovern Medical School. All procedures performed were in accordance with the ethical standards of the institution of the first and second authors and with the Declaration of Helsinki as revised in 2000. Informed consent was obtained from all individual participants included in the study.

### 2.3. Background Questionnaire

The background questionnaire sought demographic information (e.g., household income, parent's age, education level, and siblings). A modified version of Kuppuswamy's socioeconomic scale [46] was used to create a socioeconomic status (SES) score based on parental education, occupation status, and household income. In summary, educational attainment of each parent was scored from 1 to 3, indicating the following: 1) junior high or high school diploma, 2) some college (e.g., junior college), and 3) college and above. The mean of the mother and father's educational attainment scores was used as a parental education score. The occupational status of each parent was scored from 1 to 3, indicating the following: 1) not in labor force, 2) part-time worker, and 3) full-time worker. Similarly, the mean of the mother and father's occupational status was used as a parental occupation score. If the mother was single, only her scores were used to calculate the SES score. The total household income was scored from 1 to 3 for income levels under $25 k, between $25 k and 75 k, and $75 k and above, respectively, based on categories derived from the U.S. Census. As for the Japanese total household income, it was scored from 1 to 3 based on the income levels of under 2500 k yen, between 2500 k–7500 k yen, and 7500 k yen and above. Finally, the total SES score was created by adding the scores from parental education, occupational status, and household income—a range between 3 (minimum) to 9 (maximum). The participants were grouped as high SES (SES score ≥7) and low SES (SES score <7) using the median of SES scores as the cutoff.

### 2.4. Parenting Stress Index (PSI)

Mothers' parenting stress was measured using the PSI [44], which is a standardized measure to evaluate stress in parent-child systems. This instrument has been used in numerous studies to examine the effect of having a child with autism on parent stress in the U.S. and Japan e.g., [5, 8]. The PSI assesses two domains of parenting stress using 101 items based on parent report on Likert-type scales ranging from strongly agree (1) to strongly disagree (5). It assesses a child domain, with high scores indicating that parenting stress originates from child temperament and characteristics that make parenting difficult, and a parent domain, with high scores indicating that the source of stress emanates from some characteristics of parents, including parent and family context factors. The Japanese version of the PSI has shown adequate internal reliability and validity [47]. Three Japanese nursing experts, including two who lived in the U.S. more than three years, translated the original PSI form and conducted a pretest with 1,109 Japanese mothers. As a result, they eliminated the questions that were difficult for Japanese mothers to answer and did not seem to reflect Japanese parenting stress upon consultation with the developer of the PSI, Richard Abidin (e.g., a question about mother's educational attainment, physical intimacy with spouse, etc.). Twenty-four items from the original version were eliminated, and one question was added in the Japanese PSI version for a total of 78 items. In addition, the scoring of subscales has been adjusted in the Japanese PSI using the factor analysis of a sample tested after translation into Japanese. The raw score and the percentile of the parenting stress total, child domain, and parent domain (PSI, J-PSI) were used as our measure of parenting stress in the current study. The critical cutoff score for high stress is the 85^th^ percentile. The score ranges of 81–84 percentile are considered borderline, while scores in the 16–80 percentile are considered within normal limits. Because the total of items between the original and Japanese versions were different, we compared the number of people whose results were clinically significant (85^th^ percentile and above) with the number of those whose results were not clinically significant (84^th^ percentile and below) between the countries instead of comparing the scores from each country.

### 2.5. Assessment of Children's Autistic Symptom Severity

We used the SCQ and the SRS-2 to measure the severity of autistic symptoms in children. Details of these two measures are described below.

#### 2.5.1. Social Communication Questionnaire (SCQ)

SCQ [41] is a screening measure completed by caregivers to identify children who would benefit from a full autism diagnostic evaluation. It consists of 40 dichotomous items, derived from the autism diagnostic interview-revised [45]. The SCQ current (typically used for children under 4 years old, and it examines the child's behavior over the last 3 months) and lifetime (typically used for children over 4 years old, and it focuses on child's lifetime history and between the ages 4–5) versions were used for the current study. Scores above the cutoff of 15 suggest that the individual is likely to have autism, and a more extended evaluation should be undertaken. In the Japanese manual, it is explained that the developers of the SCQ, Michael Rutter, Anthony Bailey, and Catherine Lord, were consulted regarding all translation decisions [40]. For example, the Japanese SCQ manual explains that one question that asks about the child's use of pronouns was eliminated because it is common in Japanese to omit pronouns. The Japanese SCQ has been used frequently in clinical and research settings, e.g., [48, 49]. Since the Japanese version of SCQ has one question less than the original SCQ, the corrected SCQ score was calculated for Japanese participants using the following formula: adjusted Japanese SCQ score = (raw Japanese SCQ score/39) *∗* 40.

#### 2.5.2. Social Responsiveness Scale-2 (SRS-2)

The SRS-2 is a parent-completed questionnaire that can be used as a part of a diagnostic evaluation of autism, and it focuses on aspects of social communication and social reciprocity, as well as repetitive behaviors. It consists of 65 items to be completed by an adult who is familiar with the child's current behavior and developmental history [38]. Raters are asked to respond to each item using a four-point Likert scale ranging from 1 (not true) to 4 (almost always true). Raw scores are then converted into *t*-scores. There are a total of five subcategories: social awareness, social cognition, social communication, social motivation, and restricted interests and repetitive behavior. The Japanese version is identical to the original version and has shown adequate internal reliability and validity [39]. The raw score and the t-score for the five subcategories and the total score were used as our measures of autistic symptom severity in the current study.

### 2.6. Statistical Analysis

We compared the distributions of demographic and socioeconomic characteristics of the children and their parents, as well as maternal parenting stress measured by PSI scores (total, child domain, parent domain), and children's autistic symptom severity measured by SCQ and SRS-2 scores between the participants from the U.S. and Japan. We used the chi-square test to compare the proportions for categorical variables and an independent sample *t*-test or the Mann-Whitney *U* test as its nonparametric equivalent to compare the continuous variables between the two countries.

**Figure 1:**
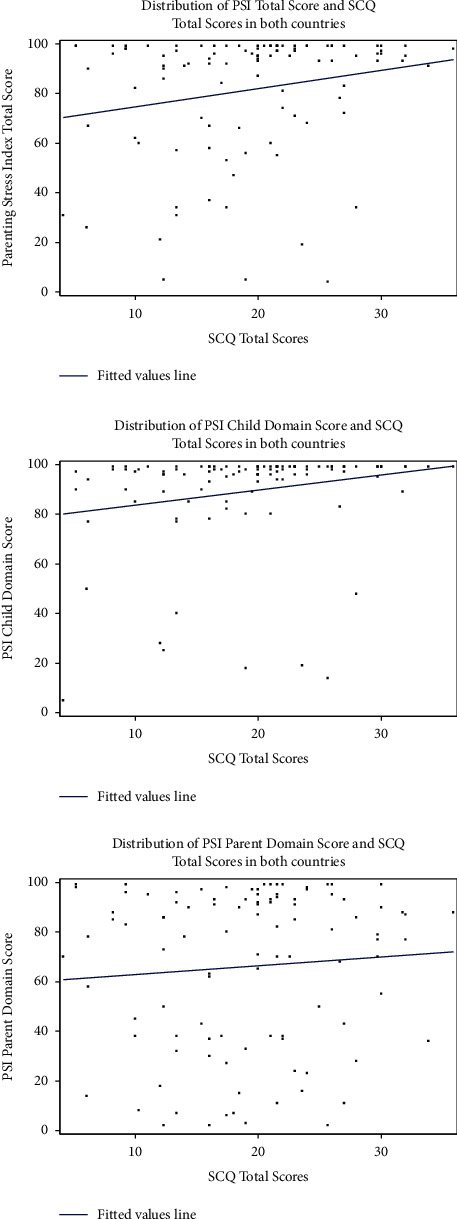
Distribution of the parenting stress index (total, child, and parent domains) and the social communication questionnaire score in combined U.S. and Japan Samples.

**Figure 2:**
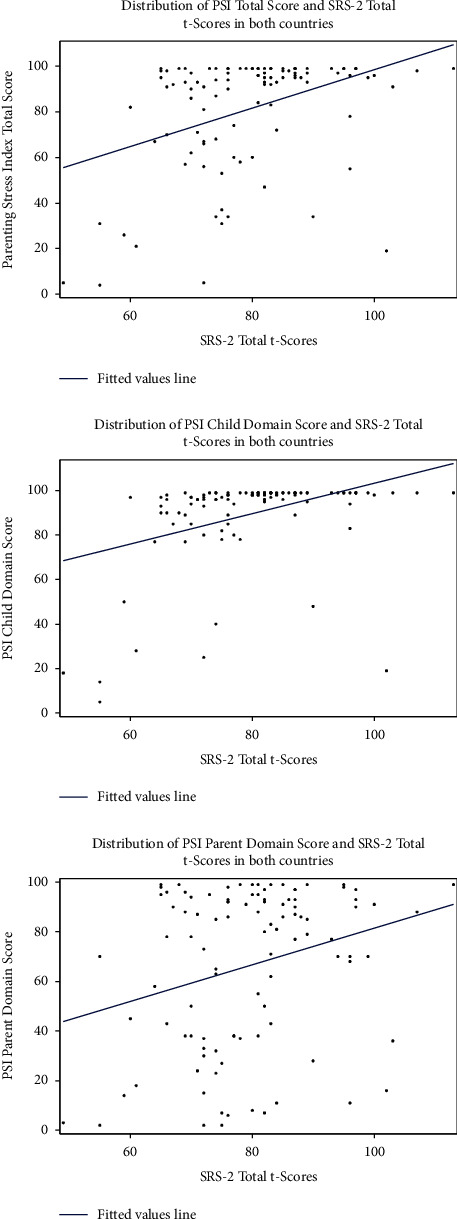
Distribution of the parenting stress index (total, child, and parent domains) and the social responsiveness scale-2 total t-scores in combined U.S. and Japan Samples.

**Figure 3:**
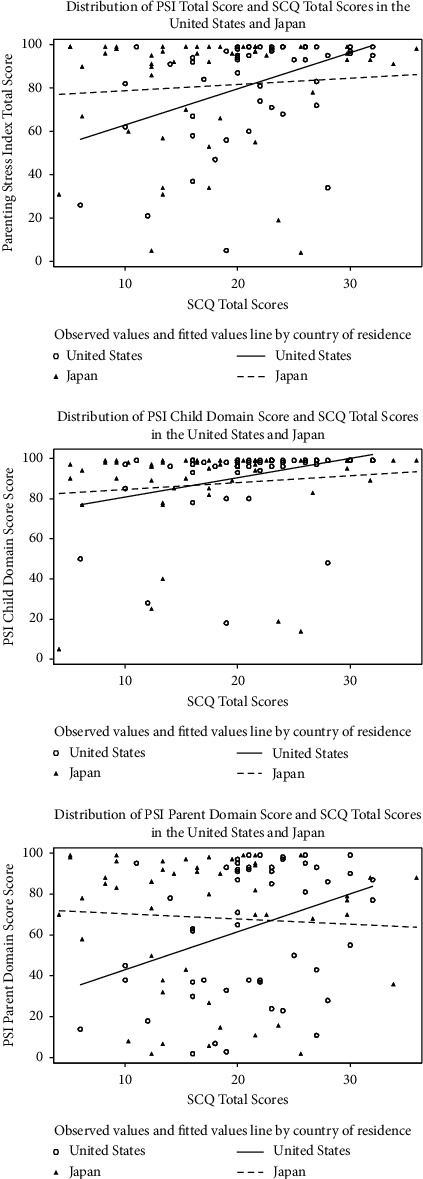
Distribution of the parenting stress index (total, child, and parent domains) and the social communication questionnaire score by country of residence (U.S. and Japan).

**Figure 4:**
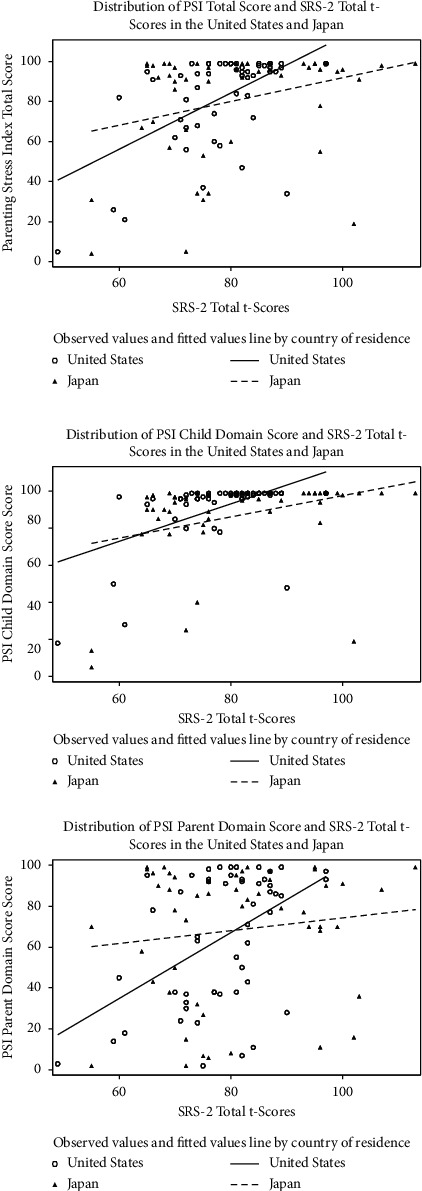
Distribution of the parenting stress index (total, child, and parent domains) and the social responsiveness scale-2 total t-scores by country of residence (U.S. and Japan).

**Figure 5:**
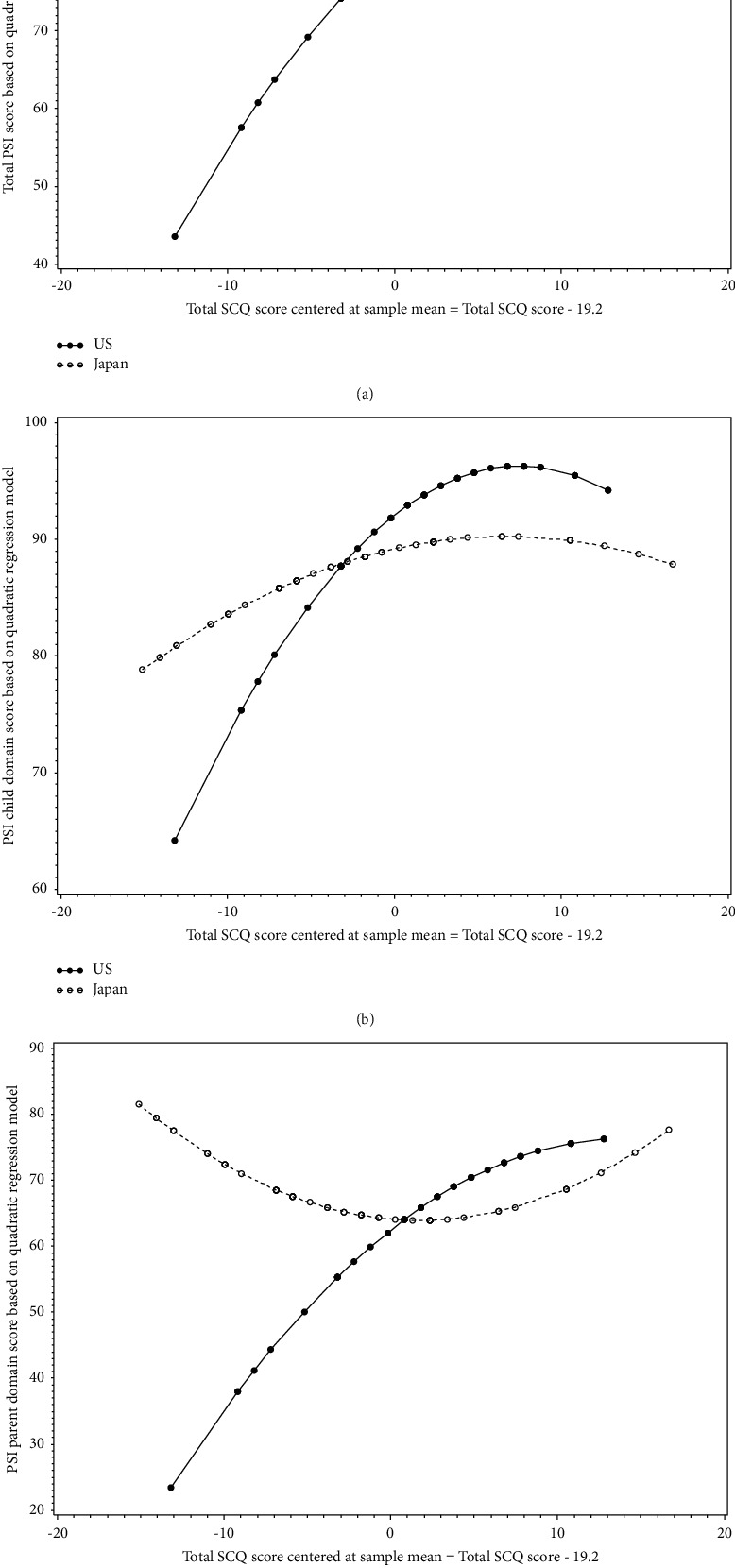
(a). Difference between the U.S. and Japan with respect to the quadratic association between parenting stress index (PSI) total score and the social communication questionnaire (SCQ) total score centered at sample mean. Note: (a) is a graphical display of the following fitted quadratic regression model: “mean (maternal stress level based on total parenting stress index score)” = “ 80.85 + 1.83 × (SCQ scores − 19.2) − 0.07 × (SCQ score − 19.2)^2^–1.68 × (Country) − 1.52 × [(SCQ score − 19.2) × Country] + 0.11 × [(SCQ score − 19.2)^2^ × Country]” *P* value for interaction between the country of residence and total SCQ score centered at sample mean = 0.08. (b). Difference between the U.S. and Japan with respect to the quadratic association between the child domain score of the parenting stress index (PSI) and social communication questionnaire (SCQ) total score centered at sample mean. Note: (b) is a graphical display of the following fitted quadratic regression model: “mean (maternal stress level based on parenting stress index child domain score)” = “92.07 + 1.13 × (SCQ scores − 19.2) − 0.07  × (SCQ  − 19.2)^2^–2.89 × (Country) − 0.80 × [(SCQ  − 19.2) × Country] + 0.05 × [(SCQ score − 19.2)^2^ × Country]” *P* value for the interaction between the country of residence and total SCQ score centered at sample mean = 0.38. (c). Difference between the U.S. and Japan with respect to the quadratic association between the parent domain score of the parenting stress index (PSI) and the social communication questionnaire (SCQ) total score centered at sample mean. Note: (c) is a graphical display of the following fitted quadratic regression model: “mean (maternal stress level based on parenting stress index parent domain score)” = “62.45 + 2.00 × (SCQ scores − 19.2) − 0.07  × (SCQ score − 19.2)^2^ + 1.68 × (Country) − 2.22 × [(SCQ scores − 19.2) × Country] + 0.13 × [(SCQ score − 19.2)^2^ × Country]”, *P* value for the interaction between the country and total SCQ score centered at sample mean = 0.049.

**Figure 6:**
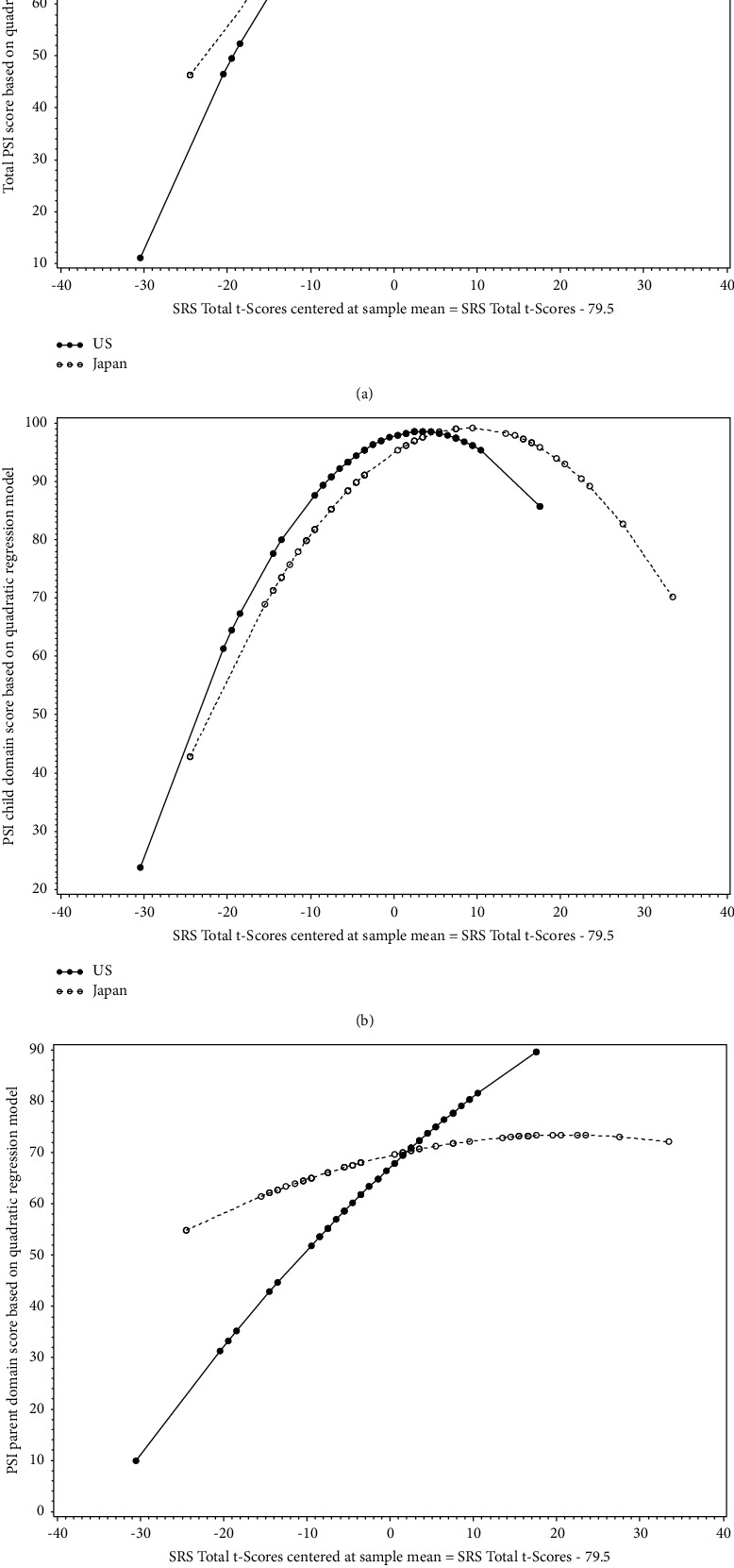
(a). Difference between the U.S. and Japan with respect to the quadratic association between the parenting stress index (PSI) total score and the social responsiveness scale-2 (SRS-2) total t-score centered at sample mean. Note: (a) is a graphical display of the following fitted quadratic regression model: “mean (maternal stress level based on parenting stress index total score)” = “87.52 + 0.97 × (SRS-2 t-score − 79.5) − 0.05 × (SRS-2 t-score  − 79.5)^2^–1.94 × (Country) − 0.15 × [(SRS-2 t-score − 79.5) × Country] + 0.02 × [(SRS-2 t-score − 79.5)^2^ × Country]”. *P* value for interaction between the country of residence and SRS-2 total t-score centered at sample mean = 0.70. (b). Difference between the U.S. and Japan with respect to the quadratic association between the child domain score of the parenting stress index (PSI) and the social responsiveness scale-2 (SRS-2) total t-score centered at sample mean. Note: (b) is a graphical display of the following fitted quadratic regression model: “mean (maternal stress level based on parenting stress index child domain score)” = “97.80 + 0.44 × (SRS-2 t-score − 79.5) − 0.06 × (SRS-2 t-score − 79.5)^2^–2.83 × (Country) + 0.47 × [(SRS-2 t-score − 79.5) × Country] + 0.01 × [(SRS-2 t-score − 79.5)^2^ × Country]” *P* value for the interaction between the country of residence and SRS-2 total t-score centered at sample mean = 0.33. (c). Difference between the U.S. and Japan with respect to the quadratic association between the parent domain score of the parenting stress index (PSI) and the social responsiveness scale-2 (SRS-2) total t-score centered at sample mean. Note: (c) is a graphical display of the following fitted quadratic regression model: “mean (maternal stress level based on parenting stress index parent domain score)” = “67.22 + 1.50 × (SRS-2 t-score − 79.5) − 0.01 × (SRS-2 t-score − 79.5)^2^ + 2.23 × (Country) − 1.12 × [(SRS-2 t-score − 79.5) × Country] + 0.004 × [(SRS-2 t-score − 79.5)^2^ × Country]” *P* value for the interaction between the country and SRS-2 total t-score centered at sample mean = 0.18.

We initially reviewed scatter plots to explore possible linear associations between maternal parenting stress (i.e., measured by PSI total, child domain, and parent domain scores) and children's autistic symptoms assessed by SCQ and SRS-2 scores, regardless of the country of residence (Figures 1 and 2, respectively). In separate scatter plots, we explored the relationship between maternal parenting stress and children's autistic symptoms assessed by SCQ and SRS-2 scores by the country of residence (Figures 3 and 4, respectively). Since the associations between PSI and either SCQ or SRS-2 scores appeared to be quadratic and the observed distributions of PSI and child's autistic severity scores by the country of residence appeared to be different, we fitted 3 separate quadratic regression models to estimate the expected mean of the PSI total, child domain, and parent domain scores based on the SCQ score of children with autism and their country of residence. Similarly, three separate quadratic regression models were fitted to estimate the expected mean of the PSI total, child domain, and parent domain scores based on the SRS-2 scores of children with autism and their country of residence. To reduce the potential multicollinearity between the linear and quadratic terms in quadratic regression models, we centered the SCQ and SRS-2 scores by subtracting the sample mean from the scores for each participant. For example, we fitted a quadratic model in which the PSI total score is a quadratic function of the SCQ score centered at a sample mean [a linear and a squared term for SCQ scores centered at the mean (herein called quadratic function)], as well as the country of residence. In addition, to capture the differences in the relationship of children's autism scores (SCQ or SRS-2) with parenting stress between the U.S. and Japan, interaction terms between children's ASD scores (SCQ or SRS-2 centered at the mean) and country of residence (US vs. Japan), as well as between squared centered ASD scores and country were kept in the model regardless of their *P*-value. Based on the estimated regression coefficients from each model, we used the equations from each model to estimate the PSI scores (total, child domain, and parent domain) for each child based on their autism scores (SCQ or SRS-2) and country of residence, and plotted the values to visualize the differences between the two countries. Additional details about all these models are provided in Figures 5(a)–5(c) and 6(a)–6(c).

To assess the statistical significance of the association between child's autism score and maternal parenting stress based on the PSI score, as differing by the country of residence, we compared models with and without interactions using a likelihood ratio test. A *P* < 0.05 from this test indicated a significant difference in the relationship of the child's autism score (SCQ or SRS-2) and PSI scores between the two countries. All statistical tests were performed at the 0.05 level of significance and conducted using SAS 9.4 statistical software [50].

## 3. Results

The majority of children with autism in the U.S. and Japan were males (78.9% vs. 76.5%, chi-2 (d*f*) = 0.08 (1), *P*=0.772) and at preschool age (44.2% vs. 39.2%, chi-2 (df) = 0.56 (2), *P*=0.754) (Table 1). A significantly larger proportion of children with autism in Japan had no siblings compared to children with autism in the U.S. (41.2% vs. 21.2%, chi-2 (d*f*) = 6.72 (2), *P*=0.032). Mothers who had a child with autism in the U.S. were significantly younger than their Japanese counterparts (mean maternal age = 37.2 vs. 41.5, t (d*f*) = −4.01 (101), *P* < 0.001). On average, compared to parents of children with autism in Japan, parents of children with autism in the U.S. had higher educational scores (median education score = 3.0 vs. 2.2, *z* = −2.43, *P*=0.017), and a larger proportion of them had high SES (73.1% vs. 47.1%, chi-2 (d*f*) = 7.27 (1), *P*=0.007, respectively). There was no significant difference between the two countries in terms of maternal parenting stress measured by PSI total, child domain, and parent domain scores. About two-thirds of mothers in both countries had PSI total scores ≥85 percentiles. Parenting stress in the child domain was even higher than the PSI total score, with more than 80% of mothers in both countries having PSI child domain scores ≥85 percentiles. Details of the characteristics of the children and their parents in the U.S. and Japan are shown in Table 1.

Overall, children with autism in the U.S. had higher SCQ scores compared to Japanese children with autism (mean SCQ score = 21.2 vs. 17.2, *t* (d*f*) = 2.94 (100), *P*=0.004), indicating that parents from the U.S. endorsed more signs of autism. Children with autism in the U.S. also had significantly higher total SRS-2 raw scores than those in Japan (mean SRS-2 raw score = 108.1 vs. 88.5, *t* (d*f*) = 4.09 (100), *P* < 0.001). However, the total SRS-2 t-scores of children with autism were not significantly different between the participants from the U.S. and Japan (mean SRS-2 *t*-score = 78.4 vs. 80.7, *t* (d*f*) = −0.99 (100), *P*=0.326), indicating that compared with their national norms, the U.S. and Japanese mothers rated their children at similar levels of severity. Similarly, there was no significant difference in the SRS-2 subdomain t-scores, except that children with autism in the U.S. had significantly higher social awareness t-scores than those in Japan (mean social awareness *t*-scores = 74.1 vs. 66.2, *t* (d*f*) = 3.25 (100), *P*=0.002). Additional details of the comparison of child autistic symptom severity measured by the SCQ and SRS-2 between the U.S. and Japan are shown in Table 2.

The distribution of the maternal PSI scores (total, child domain, and parent domain) and children's SCQ total scores (Figure 1), as well as the maternal PSI and children's SRS-2 total *t*-scores (Figure 2) showed that, regardless of the country of residence, the relationship between maternal PSI scores and children's autistic symptom severity measured by either SCQ or SRS-2 scores is nonlinear. We also observed similar nonlinear patterns for the relationship between maternal parenting stress and children's autistic symptoms based on SCQ and SRS-2 scores when looking at the distributions by country of residence (Figures 3 and 4). Findings from the separate quadratic regression models comparing the nonlinear associations between child's autistic symptom severity measured by either SCQ total score or SRS-2 total t-scores and maternal stress level (i.e., PSI total and child and parent domain scores) between the two countries are shown in Table 3. We found statistically significant difference in the quadratic association of SCQ total score with the PSI parent domain scores (*P*=0.049 for the interaction between the country of residence and SCQ total score), as well as a marginally significant difference in the quadratic association of SCQ total score with PSI total scores (Overall interaction *P*=0.08) between the U.S. and Japan. Based on the estimated regression coefficients, we obtained an equation for each fitted quadratic regression model and used that to calculate the mean PSI score (total, child domain, and parent domain) for each mother based on her child's autism characteristics measured by either SCQ total score or SRS-2 total t-scores and the country of residence. For example, based on the fitted quadratic model, the mean of the PSI total score for a mother whose child with autism has an SCQ score of 10 is 57.6 in the U.S., whereas the PSI total score is 79.4 for a mother of a child with similar autism characteristics (SCQ = 10) in Japan. In Table 4, we provided additional examples of maternal PSI total, child domain, and parent domain scores based on various SCQ or SRS-2 scores and country of residence using the fitted quadratic models. Furthermore, we provided a graphical display of the fitted nonlinear associations between children's autistic symptom severity measured by either SCQ total score (Figures 5(a)–5(c)) or SRS-2 total t-scores (Figures 6(a)–6(c)) and the PSI total score (Figures 5(a) and 6(a)), child domain score (Figures 5(b) and 6(b)), and parent domain score (Figures 5(c) and 6(c)) to facilitate a better visualization of the differences between the two countries. The graphical display of the quadratic associations between the SCQ total score and PSI total (Figure 5(a)), child domain (Figure 5(b)), and parent domain scores (Figure 5(c)) showed differences in the quadratic associations between the U.S. and Japan. However, statistically, only the differences in the quadratic association of the SCQ total score with PSI parent domain scores (Figure 5(c)) and PSI total (Figure 5(a)) were significant and marginally significant, respectively (*P*=0.049 and 0.08 for the interaction between country of residence and SCQ total score).

Similarly, it appears from the graphical display that while the quadratic association of SRS-2 total t-scores with PSI total (Figure 6(a)) and child domain (Figure 6(b)) scores are similar between the U.S. and Japan, the quadratic association of SRS-2 total *t*-scores with PSI parent domain scores is different between the two countries (Figure 6(c)). However, this difference is not statistically significant (*P*=0.18 for the interaction between country of residence and SRS-2 total t-scores).

## 4. Discussion

The main finding of our study is a nonlinear relationship between the child's autistic symptom severity and parenting stress in both countries. In addition, our findings suggest that the mothers of children with moderate levels of autism severity may report the highest levels of parenting stress. Most of the previous studies used a linear regression model to test the relationship between the child's autistic symptom severity and parenting stress [7, 51, 52] and reported inconsistent findings. In addition to differences in the study design, characteristics of the enrolled subjects, and the measures for the assessment of autistic symptom severity and parenting stress, assuming a linear relationship between a child's autistic symptom severity and parenting stress could be an explanation for inconsistent findings reported by previous studies, e.g., [12]. To the best of our knowledge, there has been only one other study that reported a nonlinear relationship between a child's autistic symptoms' severity and parenting stress. Huang et al. [13] reported a nonlinear relationship between autistic behaviors measured by the childhood autism rating scale (CARS) and parenting stress in caregivers of children with autism. Similar to Huang et al.'s study, our study found that the parents of children with mild to moderate autistic behavior problems reported higher stress. However, this association was found for stress in the parent-child relationship by Huang et al., while our study found an association with child characteristic-related stress. The nonlinear association between autism severity and parenting stress should be examined and replicated in future studies, which may shed light on the reasons for this type of association.

Both U.S and Japanese mothers showed the highest Child Domain parenting stress when their children's autistic symptoms were in the moderate range, as measured by SCQ and SRS-2 (Figures 5(b), 6(b)). Huang et al.'s [13] attributed this relationship to the pressure caregivers feel to improve their children's problems when the child has mild to moderate symptoms. By contrast, parents of children with more severe symptoms may see less likelihood of improvement, may be more likely to accept the level of disability their child displays, and may consequently blame themselves less for the child's situation, which results in less parenting stress (Huang et al.). Similarly, Da Paz et al. [53] suggested that severe autistic symptoms might facilitate the caregiver's acceptance of their children's disability, which is associated with lower depressive symptoms. In addition, it is possible that children with severe autistic symptoms appear more clearly disabled, and thus, they are not as likely to be regarded as merely undisciplined.

Another notable finding of our study was a statistically significant difference between countries in the association between parenting stress (Parent Domain) and autistic symptom severity as measured by the SCQ (Table 3 & Figure 5(c): overall *P* value for the interaction between the country and total SCQ score centered at the sample mean = 0.049). In both the scatter plot and graphical display of the fitted nonlinear model, we observed that the associations between children's SCQ scores and PSI scores for the parent domain are in opposite directions in the U.S. and Japan. Specifically, while the U.S. mothers' Parent Domain stress increased with child autistic symptom severity, Japanese mothers showed higher Parent Domain stress when children showed either low or high autistic symptom severity. One possible interpretation is that the parent's evaluation of their own functioning as a parent, which is assessed by Parent Domain stress, is influenced by the invisibility of autism. Asano et al. [35] similarly found that mothers of children with “high functioning” (less severe) autism showed higher Parent Domain parenting stress than mothers of children with “low functioning” (more severe) autism in Japan. Parents may feel shame and lose confidence when being falsely judged by others as not effectively disciplining their child. This inclination is more severe in the Asian cultural context [35, 54] than in Western cultures. On top of this, when children demonstrate severe autistic traits, mothers may feel stigmatized due to the collectivistic cultural orientation, in which being different is threatening to their identity [20].

The SRS-2 total score for the Japanese sample (mean = 88.5) was similar to that of a Japanese nationally representative sample of 257 children with autism age 6–15 mean (mean = 87.3) [55]. The SRS-2 total mean score of our participants from the U.S. (mean = 108.1) was also similar to that of the SRS-2 total score from a large sample of 2,720 children with autism ages 3–17 in the U.S. (mean = 110.32) [56]. As for the SCQ, there are not many Japanese studies that have used the Japanese version of the SCQ to assess children's autistic symptom severity. A recent study conducted by Fujino et al. [57] reported the median SCQ score of school-aged children with autism was 19, which did not differ much from our Japanese sample (mean = 17.2). Our U.S. SCQ mean score was 21.2, which was also not much different from the mean SCQ score reported by a recent U.S. study of parents of school-aged children with autism (mean = 22.17) [58].

Japanese children's lower SRS-2 and SCQ scores may be related to a culturally conditioned tendency for their mothers to use the mid-point response style (MRS), whereas the U.S. mothers might be more likely to use an extreme response style (ERS) [59, 60] when filling out questionnaires. Chen et al. explained these cross-cultural differences as associated with the cultural orientation of individualism in the West and collectivism or interdependency in Asia. In Asian countries, it is preferable to avoid expressing one's own opinion clearly to keep harmony with others. Thus, responses are likely to be neutral and ambiguous [59,60]. A similar tendency toward the cultural bias of parents' rating toward the mean was also found in an evaluation of psychometric properties in the SRS-Korean version [61]. If this is so, then mothers from Japan in our study may have tended to express more neutral and less extreme evaluations of their children compared to mothers from the U.S., who may have been more focused on differences between their children and neurotypical children.

In this study, we found a cross-cultural difference in the relationship between Parent Domain stress and the SCQ, but not the SRS-2. Previous studies have reported concurrent validity between the SCQ and SRS-2 scores e.g., [62]. However, our Japanese sample demonstrated a reverse *u*-shaped curve for the relationship between parenting stress and autistic symptom severity with the SCQ, which was not found for the SRS-2. The discrepancy may be attributable to the content of the measures; the SCQ focuses on autism-related behaviors that are usually seen in early childhood, while SRS-2 also measures aspects of autism-related behaviors that may be seen at more developmentally advanced levels.

This study had several limitations. First, it had a relatively small sample size, and therefore, the results should be interpreted with caution. However, the demographic characteristics of our samples indicated in Table 1 were consistent with the differences in national averages in the U.S. and Japan (The average childbearing age for the U.S. and Japanese females in 2015 was 28.73 and 30.84, respectively [63, 64]. The average length of female schooling for U.S. females in 2015 was 13.4 years and 12.6 years for Japanese females [65]. The mean household income in the U.S. was $59,556 and in Japan was $41,194 (United Nations Development Program). Although we are unable to find comparable data for employment rates for mothers of children between 2 and 12, the female employment rate in the U.S. was 53.7% and 48% in Japan ([66, 67]). Future work can benefit from investigating our observed differences with different populations and a larger sample size. Another limitation is that our study relies on data from maternal self-reports. Data collected from self-reports may reflect not only cultural differences between Japan and the U.S. related to mothers' perception and evaluation of their children but also their willingness to report. Assessments of autistic symptom severity using other measures, such as reports by teachers or observation, may complement mothers' reports in future studies. Previous studies have found a moderate correlation between the teacher and parent ratings of SRS-2 and SCQ scores of children with autism, including a study conducted in Japan [55,62,68]. Finally, as seen in much research with volunteer participants, our study has a potential sampling bias. It is possible that mothers who have the time available to participate in research and are also actively involved in finding help for their children with autism were overrepresented in our sample.

Despite some limitations, this study is one of only a few that has investigated cultural differences associated with the relationship between child autistic symptoms and parenting stress among mothers of children with autism. To our knowledge, this is the first study that used a Japanese sample to examine the influence of culture on child autistic symptoms and parenting stress. In addition, our study has implications for future research on the examination of child characteristics and parenting stress using a nonlinear model. Our findings also suggest that parents need support regardless of autism severity in children, including those with mild to moderate symptoms. Expanding on this line of research and understanding cultural influences on parenting stress may help service providers and agencies offer more culturally sensitive services, parent-education courses, and intervention programs. Continued studies of this cross-cultural topic in other populations are needed.

## Figures and Tables

**Table 1:** Comparison of child and parent characteristics in the U.S. and Japan.

Variable	United States (*n* = 52)^a^	Japan (*n* = 51)	*Test statistic* ^*c*^ *(df)*	*P* value
Children				
Gender			0.08 (1)	0.772
Male	41 (78.9)	39 (76.5)		
Female	11 (21.2)	12(23.5)		
Age			0.56 (2)	0.754
Preschool (0–5.5 years)	23 (44.2)	20 (39.2)		
Lower middle childhood (5.5–9 years)	17 (32.7)	16 (31.4)		
Upper middle childhood (9.1–12 years)	12 (23.1)	15 (29.4)		
Number of Sibling			6.72 (2)	0.034
0	11 (21.2)	21 (41.2)		
1	25 (48.1)	23 (45.1)		
2 or more	16 (30.8)	7 (13.7)		
Parent				
Maternal age (years), (Mean ± SD)	37.2 ± 5.6	41.5 ± 5.3	−4.01 (101)	<0.001
Household Income level^b^			2.37 (2)	0.299
Low (<$25 k or <2500 k yen)	4 (7.7)	4 (7.8)		
Medium ($25 k-$75 k or 2500 k–7500 k yen)	20 (38.5)	27 (52.9)		
High (> $75 k or 7500 k yen)	28 (53.8)	20 (39.2)		
Parental educational score, Median ± IQR	(3.0 ± 0.5)	(2.2 ± 1.0)	−2.43^c^	0.017
Parental occupational score, Median ± IQR	(2.3 ± 1.00)	(2.0 ± 0.5)	−1.99^c^	0.049
SES based on socioeconomic score			7.27 (1)	0.007
Low (SES score <7)	14 (26.9)	27 (52.9)		
High (SES score ≥7)	38 (73.1)	24 (47.1)		
Maternal Stress measured by PSI score, Mean ± SD				
Total Score	82.1 ± 23.6	80.9 ± 27.1	0.24 (101)	0.812
≥85 percentile	33 (63.46)	36 (70.59)		
<85 percentile	19 (36.54)	15 (29.41)		
Child domain	91.7 ± 17.4	87.00 ± 23.3	1.16 (101)	0.248
≥85 percentile	45 (86.54)	41 (80.39)		
<85 percentile	7 (13.46)	10 (19.61)		
Parental Domain	64.5 ± 32.0	68.5 ± 32.1	−0.63 (101)	0.527
≥85 percentile	24 (46.15)	24 (47.06)		
<85 percentile	28 (53.85)	27 (52.94)		

*Note.* Data are reported as frequencies (percentages), otherwise as indicated, IQR: interquartile range, df: degree of freedom, SES: socioeconomic status, and PSI: parenting stress index. ^a^It included 38 non-Hispanic white American, six Hispanic/Latino Americans, six Black/African Americans, one Middle Eastern American, and one with no answer. ^b^The three levels of Japanese income were created based on the Japanese yen to U.S. dollars currency exchange (1 dollar = 111 yen from 2014 to 2017). Because our income question answers only contain numbers for every $25 k (or 2500 k yen), we decided to calculate the Japanese income levels as 1 dollar = 100 yen. ^c^Z-score from Mann–Whitney Wilcoxon Test. ^c^Based on t-value for the comparison of continuous variables, and chi-2 value for the comparison of categorical variables.

**Table 2:** Comparison of child ASD characteristics measured by social communication questionnaire (SCQ) and social responsiveness scale-2 (SRS-2) between the U.S. and Japan.

Variable	United States (*n* = 52)	Japan (*n* = 51)	*t-value (df)*	*P* value
Social Communication Questionnaire (SCQ)^a^				
Total Score	21.2 ± 5.8	17.2 ± 7.8	2.94 (100)	0.004
Social Responsiveness Scale-2 (SRS-2)^b^				
Total raw score	108.1 ± 23.4	88.5 ± 24.9	4.09 (100)	<0.001
Total *t*-score	78.4 ± 9.3	80.7 ± 13.8	−0.99 (100)	0.326
Social Awareness	14.1 ± 3.8	10.9 ± 3.7	4.28 (100)	<0.001
Social Awareness *t*-score	74.1 ± 11.7	66.2 ± 12.8	3.25 (100)	0.002
Social Communication	36.6 ± 9.0	29.8 ± 9.5	3.71 (100)	<0.001
Social Communication *t*-score	77.0 ± 10.1	79.1 ± 14.0	−0.87 (100)	0.390
Social Cognition	20.5 ± 5.1	17.5 ± 4.9	3.08 (100)	0.003
Social Cognition *t*-score	75.1 ± 9.6	75.4 ± 11.8	−0.15 (100)	0.879
Social Motivation	15.98 ± 5.42	13.08 ± 5.31	2.73 (100)	0.008
Social Motivation *t*-score	69.8 ± 10.7	67.1 ± 14.8	1.07 (100)	0.290
Restricted Interest and Repetitive Behavior	20.90 ± 6.27	17.26 ± 6.94	2.78 (100)	0.006
Restricted Interest and Repetitive Behavior *t*-score	79.0 ± 13.6	85.1 ± 17.7	−1.95 (100)	0.054
DSM-5 Compatible Subscales				
Social Communication and Interaction	87.2 ± 18.9	71.2 ± 19.0	4.24 (100)	<0.001
Social Communication and Interaction *t*-score	77.1 ± 9.2	77.6 ± 12.7	−0.22 (100)	0.826

*Note.* Data are reported as mean and standard deviation, otherwise as indicated. df: degree of freedom. ^a^Japanese version of SCQ has one question less than the original SCQ. Since each question will be scored as 0 or 1, corrected SCQ score was calculated for Japanese participants using the following formula: adjusted Japan SCQ score = (raw Japanese SCQ score/39) × 40. ^b^ Data missing for one Japanese participant.

**Table 3:** Curvilinear association of maternal parenting stress level (parenting stress index) with child ASD characteristics (social communication questionnaire and social responsiveness scale-2 scores centered at sample mean) by country of residence.

	Maternal stress														
	Total score					Child domain score					Parent domain				
	(SE)	*t* value(df = 1)	*P*	Overall Interaction^a^		(SE)	*t* value (df = 1)	*P*	Overall Interaction ^a^		(SE)	*t* value (df = 1)	*P*	Overall Interaction^a^	
				chi-2 (df = 2)	*P*				chi-2 (df = 2)	*P*				chi-2 (df = 2)	*P*
SCQ score ^b^ centered at sample mean (SCQ Score-C) = SCQ score–19.2															
Intercept	80.85 (4.47)	18.07	<0.01			92.07 (3.68)	25.02	<0.01			62.45 (5.69)	10.97	<0.01		
SCQ Score-C	1.83 (0.62)	2.94	<0.01			1.13 (0.51)	2.20	0.03			2.00 (0.79)	2.52	0.01		
(SCQ Score-C)^2^	-−0.07 (0.08)	−0.98	0.33			−0.07 (0.06)	−1.18	0.24			−0.07 (0.10)	−0.73	0.46		
Country	−1.68 (6.50)	−0.26	0.80			−2.89 (5.35)	−0.54	0.59			1.68 (8.27)	0.20	0.84		
Country × SCQ Score-C	−1.52 (0.77)	−1.98	0.050	4.94	0.08	−0.80 (0.63)	−1.27	0.21	1.95	0.38	−2.22 (0.98)	−2.27	0.02	6.00	0.049
Country × (SCQ Score-C)^2^	0.11 (0.09)	1.23	0.22			0.05 (0.07)	0.68	0.50			0.13 (0.12)	1.15	0.25		
SRS-2 *t*-score centered at sample mean (SRS-2 *t*-Score-C) = SRS-2 *t*-score –79.5															
Intercept	87.52 (3.60)	24.31	<0.01			97.80 (2.64)	37.10	<0.01			67.22 (4.95)	13.58	<0.01		
SRS-2 *t*-Score-C	0.97 (0.39)	2.45	0.02			0.44 (0.29)	1.55	0.12			1.50 (0.54)	2.77	<0.01		
(SRS-2 *t*-Score-C)^2^	−0.05 (0.02)	−2.17	0.03			−0.06 (0.02)	−3.81	<0.01			−0.01 (0.03)	−0.39	0.70		
Country	−1.94 (5.57)	−0.35	0.72			−2.83 (4.08)	−0.69	0.49			2.23 (7.65)	0.29	0.77		
Country × SRS-2 t-Score-C	−0.15 (0.47)	−0.32	0.75	0.70	0.70	0.47 (0.34)	1.37	0.17	2.18	0.33	−1.12 (0.65)	−1.74	0.08	3.42	0.13
Country × (SRS-2 *t*-Score-C)^2^	0.02 (0.03)	0.66	0.51			0.01 (0.02)	0.75	0.45			0.004 (0.04)	0.09	0.93		

*Note*. Regression coefficients from the quadratic regression model, SE: standard error of quadratic regression coefficients, df = degree of freedom, SRS: social responsiveness scale, and SCQ: social communication questionnaire. ^a^Based a likelihood ratio test that compared models with and without interactions terms. ^b^Japanese version of SCQ has one question less than the original SCQ. Since each question has a binary response (i.e., scored as 0 or 1), the corrected SCQ score was calculated for Japanese participants using the following formula: adjusted Japan SCQ score = (raw Japanese SCQ score/39) × 40.

**Table 4:** Maternal parenting stress level based on mean parenting stress index (PSI) score and various examples of social communication questionnaire (SCQ) total scores and social responsiveness scale-2 (SRS-2) total T-scores by the country of residence using an interactive quadratic model.

Country	U.S.	Japan	U.S.	Japan	U.S.	Japan
Maternal stress	Mean PSI total score	Mean PSI total score	Mean PSI child domain score	Mean PSI child domain score	Mean PSI parent domain score	Mean PSI parent domain score
SCQ score ^a^ centered at sample mean = SCQ score−19.2						
10–19.2 = −9.2	57.6	79.4	75.4	84.2	37.9	71.4
15–19.2 = − 4.2	71.8	78.5	86.0	87.4	52.8	66.1
20–19.2 = 0.8	82.3	79.4	92.9	89.4	64.0	64.0
25–19.2 = 5.8	88.9	82.2	96.1	90.2	71.6	65.0
30–19.2 = 10.8	91.9	86.8	95.5	89.8	75.6	69.0
SRS-2 total t-score centered at sample mean = SRS-2 total t-score−79.5						
60–79.5 = −19.5	49.5	57.5	64.4	58.3	33.3	58.7
70–79.5 = −9.5	73.8	74.9	87.7	81.8	51.9	65.1
80–79.5 = 0.5	88.0	86.0	98.0	95.4	68.0	69.6
90–79.5 = 10.5	92.1	90.6	95.3	99.1	81.6	72.4
100–79.5 = 20.5	86.1	89.0	79.6	93.0	92.7	73.4

SCQ: social communication questionnaire, SRS-2: social responsiveness scale-2, and PSI: parenting stress index. ^a^Japanese version of SCQ has one question less than the original SCQ. Since each question will be scored as 0 or 1, the corrected SCQ score was calculated for Japanese participants using the following formula: adjusted Japan SCQ score = (raw Japanese SCQ score/39) × 40.

## Data Availability

The data that support the findings of this study will be available from the corresponding author upon reasonable request when the study publications are complete.
